# Age Estimation through Hounsfield Unit Analysis of Pelvic Bone in the Romanian Population

**DOI:** 10.3390/diagnostics14182103

**Published:** 2024-09-23

**Authors:** Emanuela Stan, Alexandra Enache, Camelia-Oana Muresan, Veronica Ciocan, Stefania Ungureanu, Alexandru Catalin Motofelea, Adrian Voicu, Dan Costachescu

**Affiliations:** 1 Department of Neuroscience, Discipline of Forensic Medicine, Bioethics, Deontology and Medical Law, “Victor Babes” University of Medicine and Pharmacy, 300041 Timisoara, Romania; emanuela.stan@umft.ro (E.S.); enache.alexandra@umft.ro (A.E.); muresan.camelia@umft.ro (C.-O.M.); veronica.luta@umft.ro (V.C.); stefania.ungureanu@umft.ro (S.U.); 2 Ethics and Human Identification Research Center, Department of Neurosciences, “Victor Babes” University of Medicine and Pharmacy, 300041 Timisoara, Romania; 3 Institute of Legal Medicine, 300610 Timisoara, Romania; 4 Department of Internal Medicine, “Victor Babes” University of Medicine and Pharmacy, 300041 Timisoara, Romania; 5 Department II, Pharmacology-Pharmacotherapy Discipline, Faculty of Pharmacy, “Victor Babes” University of Medicine and Pharmacy, 300041 Timisoara, Romania; adrian.voicu@umft.ro; 6 Department of Orthopedics-Traumatology, Urology, Radiology and Medical Imaging, Discipline of Radiology and Medical Imaging, “Victor Babes” University of Medicine and Pharmacy, 300041 Timisoara, Romania; costachescu.dan@umft.ro; 7 Radiology Laboratory, Emergency Municipal Clinical Hospital, 300054 Timisoara, Romania

**Keywords:** forensic anthropology, computed tomography, Hounsfield unit, age and sex estimation, os coxae

## Abstract

**Background**: Bone density is affected by age- and sex-related changes in the os coxae, often known as the pelvic bone. Recent developments in computed tomography (CT) imaging have created new opportunities for quantitative analysis, notably regarding Hounsfield Units (HU). **Objectives**: The study aims to investigate the possibility of using HU obtained from os coxae CT scans to estimate age in the Romanian population. **Methods**: A statistical analysis was conducted on a sample of 80 pelvic CT scans in order to find any significant correlation between age, sex, and variation in density among the different pelvic bone locations of interest. According to the research, pelvic radiodensity measurements varied significantly between male and female participants, with men having greater levels. This technique may be valuable for determining an individual’s sex precisely, as evidenced by the substantial association found between HU levels and changes in bone density associated with sex. **Results**: The analysis of variance underscores that HU values exhibit a significant negative relationship with radiodensity, with a general trend of decreasing HU with increasing age. The equation derived from the ordinary least squares OLS regression analysis can be used to estimate the age of individuals in the Romanian population based on their HU values at specific pelvic sites. **Conclusions**: In conclusion, the application of HU analysis in CT imaging of the coxae represents a non-invasive and potentially reliable method for age and sex estimation, and a promising avenue in the field of human identification.

## 1. Introduction

Assessing biological profiles, including age, sex, ancestry, and stature, is a major challenge in forensic anthropology and a key element for the identification of skeletal remains [1]. In addition to ancestry and stature, establishing sex and age at death represents an essential step in establishing identity from skeletal remains in the personal identification process [2]. The assessment of biological profiles can help narrow down potential matches from missing person databases and contribute to the overall identification of missing persons. In past decades, there has been a constant focus in forensic anthropology on developing various methods of estimating age and sex from different human skeletal remains [3]. There are several methods for age and sex estimation of skeletal remains based on macroscopic analysis. 

Several studies employed qualitative techniques to estimate chronological age based on specific skeletal features; the most often employed features include the ribs, the pubic symphysis, and the auricular surface [4,5,6]. 

Several osteometric techniques for determining sex have been devised, including measurements of bones. They frequently focus on some specific population. For instance, Kranioti and Michalodimitrakis [7] designed a specific population equation for sex determination using measurements of the humerus. 

Forensic anthropology has a potential new direction with the use of Hounsfield Unit (HU) analysis in computed tomography (CT) imaging of the coxae. Bone mineral density is measured using medical imaging techniques like CT or dual-energy X-ray absorptiometry (DEXA) (bone densitometry), which gives forensic anthropologists important information for determining the age of skeletal remains. Through the examination of trabecular bone density in certain regions, such as the pubic symphysis, scientists seek to establish a relationship between variation in bone density and the determination of chronological age. Furthermore, the use of HU for age and sex estimation is currently limited, and only a small number of research has attempted to demonstrate an association between bone density and age or sex assessment. Ford et al. [8] focused on creating a new formula that employs the HU value from CT images of the proximal femur to estimate age and sex.

CT is a non-invasive method that effectively penetrates soft tissues while maintaining the integrity of the deceased. It is, therefore, appropriate for imaging skeletal remains as well as living individuals [9]. This has the potential to revolutionize the field of forensic anthropology by providing a less invasive and more accurate approach to age estimation.

In recent years, due to the growing number of immigrants in several European nations, forensic medicine has encountered significant challenges in estimating the age of living individuals [10,11]. Developing novel imaging techniques that could be utilized for sex and age estimation in living individuals is essential, even though it is simpler to employ existing approaches in the case of skeletal remains. Promising outcomes have been observed when estimating age in living people using CT scans.

Exploring the utility of HU values derived from CT scans of the os coxae in estimating sex and age is a subject of considerable interest. This is the first study investigating the correlation between sex, age, and the HU from CT scans of the pelvis in the Romanian population.

## 2. Materials and Methods

Data collection: 

The data consisting of images from computed tomography of the pelvis were collected from living adults who underwent an examination in the Radiology Department of the Municipal Emergency Hospital in Timisoara between 2020 and 2022. All the data were anonymized, except for age and sex, and collected following the Declaration of Helsinki. Informed consent was taken from all the patients, and the study was approved by the Committee on Research Ethics of the Victor Babes University of Medicine and Pharmacy Timisoara (approval No. 45/11.12.2019).

The sample consisted of 80 pelvic CT scans, subdivided into 40 females and 40 males. Each sex group mirrored the other regarding average ages and ranges within every decade from 20 to 100 years. All the scans were carried out on a Siemens Somatom Definition Edge (Erlangen, Germany) at 120 kV, 250 mAs, with a slice thickness of 0.6 mm, a 1-mm slice increment, and a B30f reconstruction algorithm. CT scans from patients with signs of pelvic trauma and those with hip replacements or other materials that could cause artifacts were not included in the study. 

**Figure 1:**
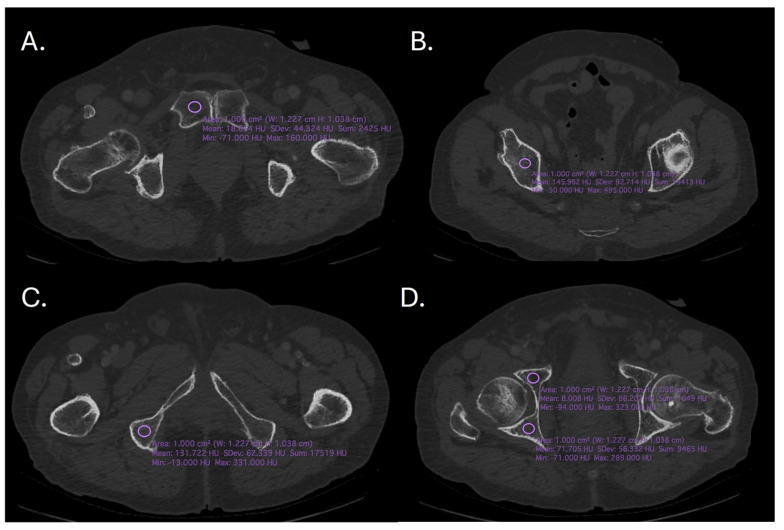
The CT images showing the region of interest (**A**). Right pubic symphysis, (**B**). Supracetabular (**C**). Ischial tuberosity, (**D**). Anterior and posterior acetabulum.

Using OsiriX software version 11.0 (Pixmeo SARL, Bernex, Switzerland), the HU measurements were carried out on an axial plane on the left and right sides of five regions of interest (ROI). The radiodensity of an object or tissue was represented on a scale called the Hounsfield Unit. The scale has a value of 0 HU for water and goes from −1000 (air) to +1000 HU (dense bone). Measuring and analyzing the HU values in certain areas of interest that correspond to a 1 cm^2^ circle was the aim of OsiriX software analysis (Figure 1). The following areas of interest were examined in this study: the left (PI) and right (Pr) sides of the pubic symphysis; the anterior acetabulum on the left (Aal) and right (Aar) sides; the posterior acetabulum on the left (Pal) and right (Par) sides; the acetabular plate on the left (Apl) and right (Apr) sides; and the ischial tuberosity on the left (Itl) and right (Itr) sides.

Statistical analysis: 

We conducted a series of statistical analyses to explore the relationships between age, sex, and bone radiodensity measured in Hounsfield Units (HU). First, we performed descriptive statistics to summarize key variables, including means, standard deviations, and proportions for continuous and categorical data.

A power sample size calculation was performed previously, ensuring at least 80% power with a 95% confidence interval.

To assess differences between sexes, we used independent t-tests for HU comparisons and ANOVA for multiple site comparisons, assuming Gaussian distribution.

Correlation analysis was performed using Pearson’s correlation coefficients to evaluate the association between age and HU values across different pelvic sites. We then developed regression models, including Ordinary Least Squares (OLS) and polynomial regression, to predict age from HU values and sex. Model fit was assessed using R-squared and adjusted R-squared values.

We conducted sex-specific regression analyses to determine if the relationship between age and radiodensity differs by sex, including interaction terms where appropriate.

Finally, we evaluated model performance using adjusted R-squared, F-statistics, and residual diagnostics. Model selection was further informed by the Akaike Information Criterion (AIC) and Bayesian Information Criterion (BIC). The threshold for statistical significance was set at a *p*-value < 0.05. All statistical analyses were conducted using Python version 3.12.

## 3. Results

Table 1 displays the mean and standard deviation of radiodensity measurements for each site. Based on an analysis of HU values between the two groups, males generally exhibited higher HU values at various pelvic sites than females. 

Further analysis systematically evaluates the symmetry and variability in pelvic radiodensity by comparing HU measurements across key pelvic landmarks on the right and left sides. Key findings show no significant side-to-side differences in HU values for most locations, suggesting symmetrical radiodensity (Table 2).

The study revealed a clear trend of decreasing radiodensity with advancing age across various pelvic sites. This is evident in the gradual reduction of mean Hounsfield Unit (HU) values from younger to older age groups. There was a marked increase in the variability of radiodensity measurements in the older age groups, particularly in the 60–70 and 70–80-year ranges. This suggests greater heterogeneity in bone density among older people. Extremely high HU values in the 60–70 age group for the left ischial tuberosity indicate potential outliers or specific pathological conditions within this cohort (Table 3).

**Figure 2:**
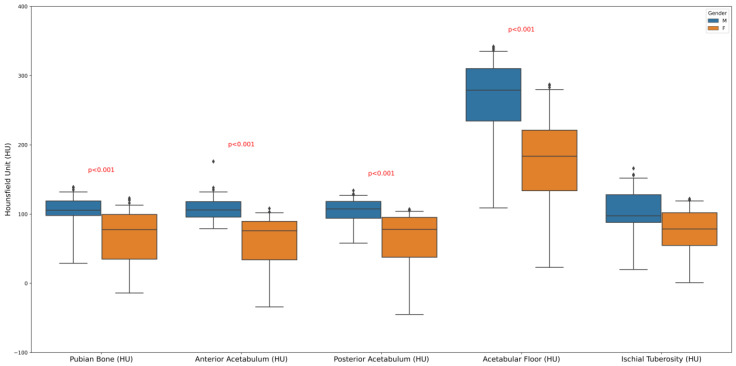
Sex differences in pelvic bone radiodensity: a boxplot analysis of Hounsfield Units.

The boxplot displays the distribution of HUs, a measure of radiodensity, across different pelvic bone regions: pubic bone, anterior acetabulum, posterior acetabulum, acetabular floor, and ischial tuberosity, with comparisons made between males and females. However, on average, the proportion of trabecular bone was marginally higher in males compared to females (Figure 2).

**Figure 3:**
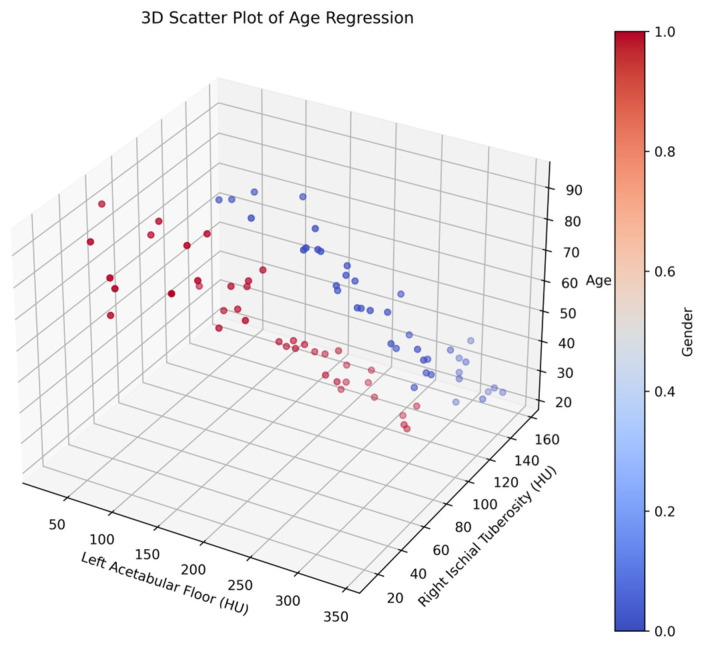
Three-dimensional scatter plot analysis of pelvic bone radiodensity: correlating age and sex (male = blue, female = red).

Figure 3 represents the three-dimensional scatter plot illustrating the multidimensional relationship between patient age, pelvic bone radiodensity, and sex. The axes demarcate the age spectrum along the vertical, and two axes represent HU measurements for the left acetabular floor and the right ischial tuberosity, which serve as proxies for bone density in specific pelvic regions. In the plot, blue points represent males and red points represent females, allowing for a clear visualization of the differences in bone density patterns between sexes.

**Figure 4:**
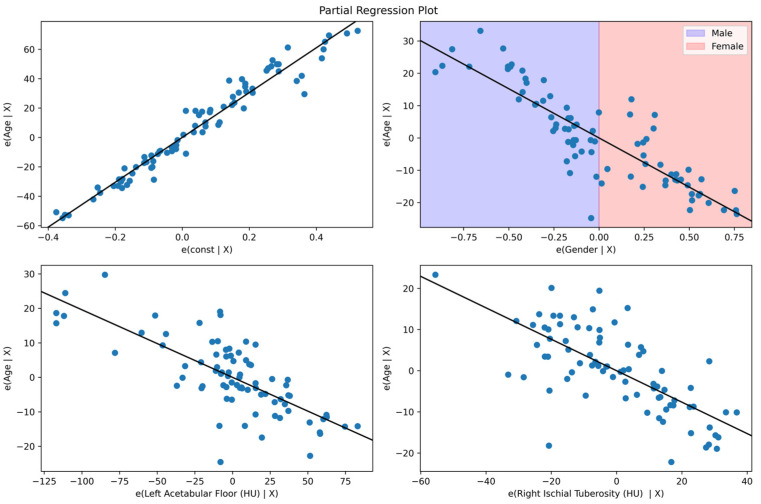
Assessing the impact of sex and age on pelvic radiodensity through partial regression.

Figure 4 shows the partial regression plots elucidating the intricate relationships between age and key pelvic bone radiodensity measures while adjusting for the multifaceted interactions with other covariates. The analyses reveal negative associations between age residuals and both the left acetabular floor and right ischial tuberosity radiodensities, suggesting age-related declines in these specific bone densities. Concurrently, it shows the impact of the sex-specific plot differential of sex on age, highlighting the nuanced interplay between these demographic factors in the context of bone health.

The coefficients derived from the regression analysis conducted in our study represent the relationship between the variables (sex, specific pelvic regions) and the Hounsfield Unit (HU) measurements. The coefficient for sex (Male–Female) is highly significant (*p* < 0.001), with a positive estimate, indicating males have higher radiodensity compared to females by about 30.44 Hounsfield Units (HU), holding other factors constant. The radiodensity of the left acetabular floor exhibits a notable negative correlation with Hounsfield Units (HU) (*p* = 0.001), indicating that a decrease in HU in this region corresponds to advancing age. Similarly, the HU value of the right ischial tuberosity also demonstrates significance (*p* < 0.001), with a negative coefficient suggesting a decline in HU with increasing age. The anticipated HU value is 122.7113. This analysis emphasizes the substantial influence of sex on pelvic radiodensity and underscores the significance of age in specific pelvic regions, with a consistent trend of declining HU values as age increases (Table 4).

OLS regression analysis was performed to generate the age estimation equation for the Romanian population, giving the following:

*Age = 152.6246 − 30.4488 × Sex − 0.1959 × HU acetabular plate (left) − 0.3820 × HU ischial tuberosity (right)*

*M = 0, F = 1*

The equation obtained through OLS regression analysis offers a means to estimate the age of individuals within the Romanian population by leveraging their HU measurements at pelvic sites.

## 4. Discussion

The development of the biological profile in forensic identification depends on two essential components: age [12,13] and sex [14,15]. The most appropriate structures for sex estimation are thought to be the pelvic bones [16]. Nonetheless, the skull can often be considered the second most reliable bone in the absence of the pelvis. According to many studies, postcranial bones can predict sex better [17]. In general, after estimating an unknown individual’s sex, one of the following stages is to approximate their age. Many techniques for estimating age have been developed using different body parts, such as teeth [18,19], pubic symphysis [20], ribs [21], and skull [22].

The comparative analysis of pelvic radiodensity between sexes and across different age groups sheds light on important factors influencing bone health and density. Here, we delve into the implications and interpretations of the findings, discussing sex disparities, age-related changes, symmetry, and potential outliers within the dataset. The statistically significant differences in HU values between male and female subjects highlighted inherent dissimilarities in bone density. Males consistently exhibited higher HU values across various pelvic sites compared to females. This observation aligns with the existing literature indicating that males typically have greater bone density than females, attributable to hormonal, genetic, and lifestyle factors. The gradual reduction in mean HU values across different age groups reflects age-related bone loss, a phenomenon well documented in the literature. The proposed method for sex estimation is comparable to other metric methods that have an accuracy range of 87.4–98.5% in which all utilize a diverse range of body parts such as pelvis and hip [15], femur [23], proximal femur [8], humerus [24,25,26], and other postcranial elements [27]. These accuracy rates are higher than those of more visual, morphometric methods, with a range spanning a high of 70% (60) and a low of 80% [28]. Notably, the older age groups exhibited greater variability in radiodensity measurements, indicative of heterogeneous bone density among the elderly population. This variability underscores the complexity of bone health management in older adults, highlighting the need for personalized approaches considering individual risk factors and comorbidities.

The correlation matrix’s significant negative correlations show that the values of Itl, Itr, and Apr tend to decline with age. Strong relationships were demonstrated by the high absolute values of Spearman’s rho (−0.825 for Itl, −0.789 for Itr, and −0.708 for Apr), and statistical significance was suggested by the low *p*-value (<0.01). This implies that age and these three factors have an inverse relationship. It is clear from examining the statistically significant variations in HU by sex that age prediction based on bone density measures has to account for these variables. A polynomial model thoroughly analyzes the complex relationship between HU values and age, making it easier to identify any potential non-linear trends (Table 5).

**Figure 5:**
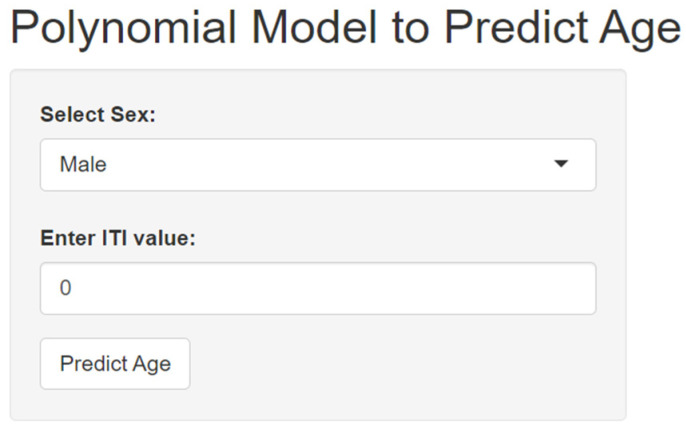
Polynomial model to predict age application.

We developed an application designed specifically for calculating age based on sex and HU values of ischiatic tuberosity (available at https://adrianvoicu.shinyapps.io/huage/) (Figure 5) (accessed on 14 July 2024). This application aimed to provide a non-invasive and accurate method for estimating age, which can be particularly useful in forensic anthropology and medical fields. Recognizing the physiological differences between sexes, this application incorporated sex-specific algorithms to tailor age calculations, enhancing accuracy. Furthermore, the interface was designed to be user-friendly, so that even individuals without a medical or scientific background can navigate and obtain accurate information about their age.

While age estimation aims to approximate the exact age at death of the individual, in practice, an estimated range is more commonly used. A recent study by Miranker [13] applied current pelvic-based age estimation techniques, including Suchey–Brooks pubic symphysis [20], Osborne auricular surface [29], Rissech acetabulum [30], and Calce acetabulum [31] aging methods on a modern population of Caucasian Americans. Miranker’s study limited accuracy to the correct inclusion of four age groups and did not use a linear regression like our current study. Still, she found a highly varied range of overall accuracy of age estimation from a very low 59.4% [31] to a high of 96.11% [30]. Age ranges were also very wide at ±19.27 [20] and relatively narrow at ±8.6 [30].

The formula derived from the OLS regression analysis provides a way to calculate the age of an individual in the Romanian population by utilizing their HU measurements at certain pelvic locations. With great potential for use in forensic investigations and archaeological research, this technique can offer a precise and non-invasive way to estimate age. This study overestimated the 40 and 90 age group of males. The 20–40 age group had 90% accuracy within a 5-year range using the male equation, whereas the 60–80 age group had over 80% accuracy. The female equation may estimate age in females aged 20–60 within a 5-year range with 80% accuracy for 20–40 and 90% accuracy for 40–60. The female sample underestimated age most at 80 and 90. This phenomenon might be attributed to lower trabecular density resulting from hormonal changes around age 50, coinciding with menopause, significantly impacting trabecular density. Furthermore, the under- or overestimation of age could be explained by patient selection bias, as the sample primarily consisted of unhealthy individuals, potentially excluding the normal population with higher bone density. Although the OLS regression analysis equation has the potential to be used for age estimation in the Romanian population, it is important to take into account its possible drawbacks and limitations before using it in a broader context. One of these constraints is the possibility of variation in pelvic morphology among various groups, which may affect the equation’s accuracy and application, especially in different populations. Moreover, the accuracy of projected ages may also be impacted by demographic differences such as age distributions or expected health issues. Furthermore, it is essential to recognize that the formula obtained using OLS regression analysis was developed with a particular sample size, indicating that it does not fully reflect the Romanian population. One major limitation of the current study is its small sample size, which makes it challenging to establish a reliable formula and accurately assess age estimation based on the available CT scans. While our study provides valuable initial insights, it is limited by its relatively small sample size of CT scans. To enhance the validity and precision of age estimation using Hounsfield Unit (HU) values, it is essential to conduct further research with a larger and more diverse dataset. Expanding the sample size will improve the age estimation formulas’ accuracy and enhance the findings’ overall reliability across different demographic groups. Validated studies with varied and larger datasets are essential to ensure the models’ dependability and refine their applicability in forensic contexts. Our study focused specifically on living individuals, and, as such, the model we developed is tailored to this context. We acknowledge that there may be limitations when applying this model to archaeological contexts or dry bones, as the conditions and variables can differ significantly. While our findings provide valuable insights into age estimation in living individuals, further research is needed to adapt or validate the model for use with dry bones in archaeological settings.

## 5. Conclusions

The results of this study demonstrate that computed tomography (CT) can be an effective tool for estimating age by analyzing Hounsfield Unit (HU) values of the os coxae. Our findings highlight that pelvic radiodensity offers valuable insights into sex differences, age-related changes, symmetry, and variability in bone density. The equation derived from the ordinary least squares OLS regression analysis can be used to estimate the age of individuals in the Romanian population based on their HU values at specific pelvic sites. However, the study also underscores the need for further research to refine age estimation models based on HU analysis of the pelvic bone, particularly within the Romanian population. This includes expanding the sample size, incorporating additional factors such as ancestry and sex, and validating models with independent datasets.

## Figures and Tables

**Table 1:** Comparative analysis of pelvic radiodensity between sexes.

	Female (N = 40)	Male (N = 40)	Total (N = 80)	*p* Value
Age				0.811 ^1^
Mean (SD)	58.9 (22.9)	60.1 (22.8)	59.5 (22.7)	
Range	22.0–92.0	23.0–93.0	22.0–93.0	
HU Pr				<0.001 ^1^
Mean (SD)	69.7 (35.4)	108.8 (18.8)	89.3 (34.4)	
Range	10.0–121.0	29.0–138.0	10.0–138.0	
HU Pl				<0.001 ^1^
Mean (SD)	64.3 (39.2)	104.2 (17.3)	84.3 (36.2)	
Range	−14.0–123.0	44.0–139.0	−14.0–139.0	
HU Aar				<0.001 ^1^
Mean (SD)	64.2 (34.8)	107.9 (15.9)	86.0 (34.8)	
Range	−20.0–103.0	81.0–138.0	−20.0–138.0	
HU Aal				<0.001 ^1^
Mean (SD)	59.0 (38.5)	106.7 (17.6)	82.8 (38.2)	
Range	−34.0–108.0	79.0–176.0	−34.0–176.0	
HU Par				<0.001 ^1^
Mean (SD)	68.7 (35.4)	107.7 (14.0)	88.2 (33.2)	
Range	−23.0–106.0	79.0–134.0	−23.0–134.0	
HU Pal				<0.001 ^1^
Mean (SD)	59.7 (42.6)	103.5 (16.8)	81.6 (39.0)	
Range	−45.0–107.0	58.0–129.0	−45.0–129.0	
HU Apr				<0.001 ^1^
Mean (SD)	179.9 (63.7)	270.2 (57.6)	225.1 (75.5)	
Range	37.0–286.0	109.0–342.0	37.0–342.0	
HU Apl				<0.001 ^1^
Mean (SD)	173.7 (65.1)	262.4 (59.7)	218.0 (76.4)	
Range	23.0–287.0	110.0–341.0	23.0–341.0	
HU ITr				<0.001 ^1^
Mean (SD)	76.6 (31.4)	107.6 (25.5)	92.1 (32.4)	
Range	15.0–122.0	73.0–157.0	15.0–157.0	
HU ITl				<0.001 ^1^
Mean (SD)	73.3 (31.5)	101.5 (29.3)	87.4 (33.4)	
Range	1.0–121.0	20.0–166.0	1.0–166.0	

^1.^Linear model Anova, Pr: pubic symphysis (right side), Pl: pubic symphysis (left side), Aar: anterior acetabulum (right side), Aal: anterior acetabulum (left side), Par: posterior acetabulum (right side), Pal: posterior acetabulum (left side), Apr: acetabular plate (right side), Apl: acetabular plate (left side), Itr: ischial tuberosity (right side), Itl: ischial tuberosity (left side). SD (standard deviation).

**Table 2:** Comparative analysis of HU values between left and right side.

	Right	Left	Total	*p* Value
HU P				0.371 ^1^
Mean (SD)	89.3 (34.4)	84.3 (36.2)	86.8 (35.3)	
Range	10.0–138.0	−14.0–139.0	−14.0–139.0	
SD P				0.020 ^1^
Mean (SD)	16.1 (5.7)	18.5 (7.6)	17.3 (6.8)	
Range	5.0–31.0	3.0–49.0	3.0–49.0	
HU Aa				0.579 ^1^
Mean (SD)	86.0 (34.8)	82.8 (38.2)	84.4 (36.5)	
Range	−20.0–138.0	−34.0–176.0	−34.0–176.0	
SD Aa				0.011 ^1^
Mean (SD)	17.6 (9.0)	14.5 (6.2)	16.1 (7.8)	
Range	6.0–64.0	2.0–38.0	2.0–64.0	
HU Pa				0.254 ^1^
Mean (SD)	88.2 (33.2)	81.6 (39.0)	84.9 (36.3)	
Range	−23.0–134.0	−45.0–129.0	−45.0–134.0	
SD Pa				0.959 ^1^
Mean (SD)	15.9 (10.3)	15.8 (8.1)	15.9 (9.3)	
Range	5.0–73.0	3.0–52.0	3.0–73.0	
HU Ap				0.558 ^1^
Mean (SD)	225.1 (75.5)	218.0 (76.4)	221.5 (75.8)	
Range	37.0–342.0	23.0–341.0	23.0–342.0	
SD Ap				0.791 ^1^
Mean (SD)	17.4 (7.3)	17.1 (7.6)	17.3 (7.4)	
Range	4.0–52.0	4.0–43.0	4.0–52.0	
HU IT				0.373 ^1^
Mean (SD)	87.4 (33.4)	92.1 (32.4)	89.8 (32.9)	
Range	1.0–166.0	15.0–157.0	1.0–166.0	
SD IT				0.462 ^1^
Mean (SD)	17.8 (11.0)	16.6 (8.5)	17.2 (9.8)	
Range	4.0–95.0	4.0–59.0	4.0–95.0	

^1.^Linear model Anova; P: pubic symphysis, Aa: anterior acetabulum, Pa: posterior acetabulum, Ap: acetabular plate, It: ischial tuberosity, HU = Hounsfield Unit, SD = standard deviation.

**Table 3:** Pelvic radiodensity trends across age groups: a Hounsfield Unit analysis.

	20–30 (N = 10)	30–40 (N = 10)	40–50 (N = 11)	50–60 (N = 9)	60–70 (N = 10)	70–80 (N = 10)	80–90 (N = 10)	90–100 (N = 10)	Total (N = 80)	*p* Value
HU Itl										0.442 ^1^
Mean (SD)	128.0 (20.2)	122.6 (17.2)	95.3 (12.5)	95.2 (14.0)	83.6 (12.1)	70.1 (23.9)	57.3 (31.4)	47.4 (23.9)	87.4 (33.4)	
Range	102.0–166.0	99.0–149.0	83.0–125.0	73.0–117.0	61.0–97.0	29.0–99.0	1.0–89.0	12.0–88.0	1.0–166.0	
HU Itr										<0.001 ^1^
Mean (SD)	130.5 (18.3)	129.5 (18.2)	102.2 (13.0)	93.1 (11.3)	80.6 (19.3)	75.9 (21.6)	55.0 (30.0)	69.0 (25.6)	92.1 (32.4)	
Range	107.0–156.0	106.0–157.0	86.0–134.0	79.0–116.0	54.0–117.0	23.0–96.0	15.0–89.0	26.0–106.0	15.0–157.0	
HU Pr										<0.001 ^1^
Mean (SD)	117.4 (6.8)	118.4 (12.9)	105.2 (15.2)	97.7 (16.6)	84.3 (30.4)	76.2 (38.9)	60.7 (36.2)	53.7 (34.5)	89.3 (34.4)	
Range	105.0–127.0	102.0–138.0	79.0–126.0	77.0–121.0	45.0–121.0	19.0–117.0	11.0–102.0	10.0–99.0	10.0–138.0	
HU Pl										<0.001 ^1^
Mean (SD)	112.5 (11.6)	114.6 (13.8)	96.9 (10.1)	94.7 (13.9)	80.0 (32.6)	68.9 (33.0)	59.4 (47.7)	47.0 (41.7)	84.3 (36.2)	
Range	97.0–132.0	99.0–139.0	81.0–114.0	71.0–117.0	34.0–124.0	24.0–101.0	−10.0–107.0	−14.0–103.0	−14.0–139.0	
HU Aal										<0.001 ^1^
Mean (SD)	101.7 (13.9)	104.3 (19.0)	102.8 (14.2)	100.4 (15.0)	79.6 (31.0)	64.4 (31.3)	52.6 (42.3)	56.6 (65.3)	82.8 (38.2)	
Range	82.0–123.0	81.0–132.0	78.0–120.0	72.0–118.0	23.0–118.0	8.0–96.0	−15.0–97.0	−34.0–176.0	−34.0–176.0	
HU Apl										<0.001 ^1^
Mean (SD)	300.7 (32.5)	263.8 (42.8)	254.4 (52.9)	250.0 (55.3)	204.8 (68.2)	191.8 (56.2)	164.9 (61.0)	113.3 (46.9)	218.0 (76.4)	
Range	245.0–341.0	214.0–311.0	183.0–316.0	184.0–318.0	98.0–279.0	78.0–244.0	78.0–223.0	23.0–175.0	23.0–341.0	
HU Par										<0.001 ^1^
Mean (SD)	107.1 (14.2)	107.2 (10.8)	104.0 (9.9)	106.4 (19.4)	82.6 (31.4)	77.6 (26.5)	62.6 (28.3)	58.0 (55.7)	88.2 (33.2)	
Range	89.0–134.0	95.0–124.0	84.0–121.0	75.0–128.0	43.0–128.0	17.0–99.0	15.0–89.0	−23.0–120.0	−23.0–134.0	
HU Aar										<0.001 ^1^
Mean (SD)	107.6 (16.2)	100.5 (14.0)	102.1 (16.9)	109.7 (24.0)	82.5 (32.1)	77.5 (20.8)	57.6 (32.3)	51.7 (53.7)	86.0 (34.8)	
Range	84.0–132.0	81.0–120.0	66.0–123.0	76.0–138.0	34.0–123.0	34.0–99.0	13.0–89.0	−20.0–106.0	−20.0–138.0	
HU Apr										<0.001 ^1^
Mean (SD)	300.7 (26.8)	285.4 (42.2)	257.6 (58.4)	250.0 (58.0)	201.3 (71.8)	203.7 (53.5)	177.6 (55.9)	123.4 (47.0)	225.1 (75.5)	
Range	267.0–342.0	234.0–339.0	187.0–326.0	185.0–308.0	109.0–278.0	89.0–253.0	101.0–235.0	37.0–189.0	37.0–342.0	
HU Pal										<0.001 ^1^
Mean (SD)	105.8 (12.0)	107.2 (15.6)	102.5 (13.7)	102.7 (16.5)	83.0 (25.4)	62.5 (34.9)	50.6 (43.7)	38.6 (53.5)	81.6 (39.0)	
Range	90.0–124.0	87.0–127.0	78.0–129.0	73.0–119.0	47.0–115.0	−11.0–93.0	−29.0–94.0	−45.0–106.0	−45.0–129.0	

^1.^Linear model Anova; summary of Hounsfield Unit (HU) measurements across various age groups and anatomical regions of interest in the os coxae. Mean values are presented with standard deviations (SD) and ranges; regions include the right pubic symphysis (HU Itl), supracetabular (HU Itr), ischial tuberosity (HU Pr), anterior acetabulum (HU Pl), posterior acetabulum (HU Aal), and others as labeled.

**Table 4:** Influence of age and sex on pelvic radiodensity: a statistical analysis of Hounsfield Units.

Model Coefficients–Age				
Sex Predictor	Estimate	SE	t	*p*
Intercept ^a^	122.176	2.6817	45.56	<0.001
Male–Female	30.449	2.0149	15.11	<0.001
HU Apl	−0.196	0.0200	−9.78	<0.001
HU ITR	−0.382	0.0437	−8.73	<0.001

^a^ Represents reference level. SE: standard error, M: male, F: female, Apl: acetabular plate (left side), ITR: ischial tuberosity. (right side), HU = Hounsfield Units.

**Table 5:** Polynomial model for males and females.

Polynomial Male Model:				Polynomial Female Model:			
	Age				Age		
Predictors	Estimates	CI	*p*	Predictors	Estimates	CI	*p*
(Intercept)	60.12	57.15–63.10	<0.001	(Intercept)	60.12	57.15–63.10	<0.001
ITl [1st degree]	−125.41	−144.21–−106.61	<0.001	ITl [1st degree]	−125.41	−144.21–−106.61	<0.001
ITl [2nd degree]	−16.70	−35.50–2.11	0.080	ITl [2nd degree]	−16.70	−35.50–2.11	0.080
ITl [3rd degree]	35.34	16.54–54.14	0.001	ITl [3rd degree]	35.34	16.54–54.14	0.001
ITl [4th degree]	−4.58	−23.38–14.22	0.624	ITl [4th degree]	−4.58	−23.38–14.22	0.624
Observations	40			Observations	40		
R^2^/R^2^ adjusted	0.852/0.835			R^2^/R^2^ adjusted	0.852/0.835		
Residual standard error: 9.262 on 35 degrees of freedom Multiple R-squared: 0.852, Adjusted R-squared: 0.835 F-statistic: 50.35 on 4 and 35 DF, *p*-value: 4.827 × 10^−14^				Residual standard error: 9.262 on 35 degrees of freedom Multiple R-squared: 0.852, Adjusted R-squared: 0.835 F-statistic: 50.35 on 4 and 35 DF, *p*-value: 4.827 × 10^−14^			

## Data Availability

The data presented in this study are available on request from the corresponding author. The data are not publicly available due to confidentiality reasons.
